# Supplementary figures and images for: A retrospective cohort study of clinical characteristics and outcomes of type 2 diabetic patients with kidney disease
Source: PeerJ. 2024 Feb 19;12:e16915. doi: 10.7717/peerj.16915 (PMC10883152; doi:10.7717/peerj.16915)

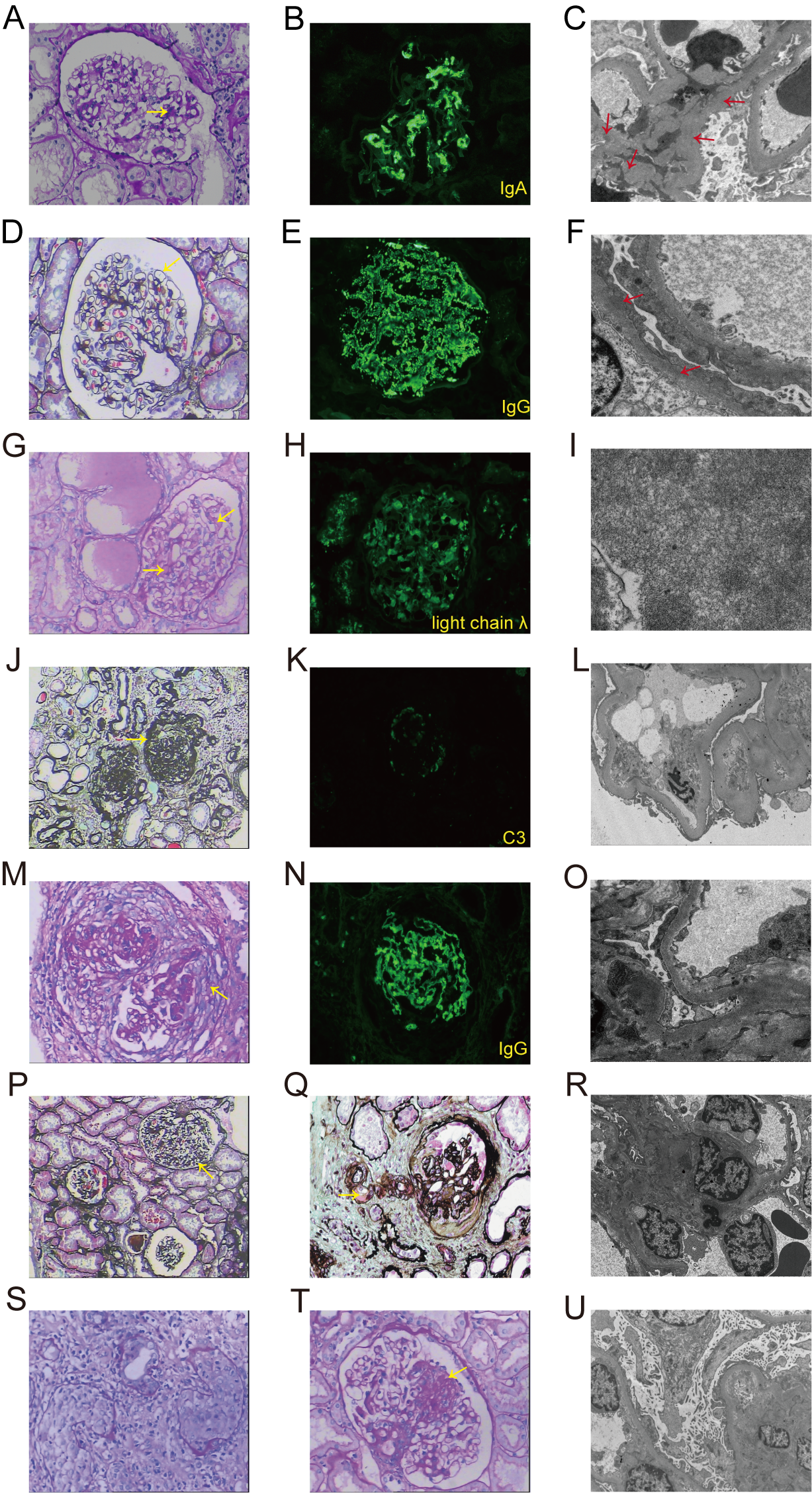

Supplement: Figure S1 — (A–C) IgA nephropathy (IgAN): light microscopy reveals mesangial matrix expansion or mesangial hypercellularity (yellow arrow). Immunofluorescence reveals the deposition of IgA in the glomerular mesangial area. Electron microscopy reveals the deposition of electron-dense material in the mesangial region (red arrow). (D–F) Membranous nephropathy (MN): light microscopy shows glomerular basement membrane thickening (yellow arrow). Immunofluorescence reveals coarse granular staining of IgG along the glomerular capillary loop and electron microscopy shows subepithelial electron-dense deposits and diffused fusion of the foot processes of podocytes (red arrow). (G–I) Kidney amyloidosis: light microscopy shows extensive deposits of pink amorphous eosinophilic material (yellow arrow). Immunofluorescence reveals deposits of λ along the mesangial glomeruli and capillary walls. Electron microscopy reveals randomly arrayed nonbranching fibrils (8–10 nm in diameter). (J–L) Sclerosing glomerulonephritis: more than 50% of the glomeruli show global sclerosis (yellow arrow). Furthermore, immunofluorescence shows non-specific C3 deposition in sclerotic areas and electron microscopy shows no electron-dense material deposition. (M–O) Crescentic glomerulonephritis: light microscopy reveals cellular crescent formation (yellow arrow). In anti-GBM positive crescentic nephritis, immunofluorescence shows linear deposition of IgG along capillary walls and electron microscopy showed no electron-dense material deposition. (P) Obesity-related nephropathy: light microscopy shows glomerulomegaly (yellow arrow). (Q–R) Hypertensive nephropathy: light microscopy reveals hyaline degeneration of glomerular arterioles and ischemic glomerular sclerosis (yellow arrow). Electron microscopy shows no electron-dense material deposition and partial fusion of the foot processes of podocytes. (S) Tubulointerstitial nephritis: light microscopy shows tubulitis and renal interstitial edema with inflammatory cell inf [file peerj-12-16915-s001.png]
